# Uptake of health services among truck drivers in South Africa: analysis of routine data from nine roadside wellness centres

**DOI:** 10.1186/s12913-017-2595-3

**Published:** 2017-09-13

**Authors:** Samanta Tresha Lalla-Edward, Sydney Ncube, Paul Matthew, Catherine A. Hankins, W.D. Francois Venter, Gabriela B. Gomez

**Affiliations:** 1 Wits Reproductive Health & HIV Institute, University of the Witwatersrand, Hillbrow Health Precinct, 22 Esselen Street, Hillbrow, 2001 Johannesburg, South Africa; 2 North Star Alliance, Durban, South Africa; 3 Department of Global Health and Amsterdam Institute for Global Health and Development, Academic Medical Centre, University of Amsterdam, Amsterdam, The Netherlands; 4 Department of Epidemiology, Biostatistics, and Occupational Health, Faculty of Medicine, McGill University, Montreal, Canada; 5 Department of Global Health and Development, London School of Hygiene and Tropical Medicine, London, UK

**Keywords:** Truck drivers, Healthcare, Service delivery, South Africa, Roadside Wellness Centre, Access, Programme

## Abstract

**Background:**

Long-distance truck drivers are occupationally susceptible to poor health outcomes. Their patterns of healthcare utilisation and the suitability of healthcare services available to them are not well documented. We report on truck driver healthcare utilisation across South Africa and characterise the client population of the clinics serving them for future service development.

**Methods:**

We analysed anonymised data routinely collected over a two-year period at nine Roadside Wellness Centres. Associations between services accessed and socio-demographic characteristics were assessed using univariable and multivariable logistic regression models.

**Results:**

We recorded 16,688 visits by 13,252 individual truck drivers (average of 1.26 visits/person) who accessed 17,885 services for an average of 1.07 services/visit and 1.35 services/person. The mean age of truck drivers was 39 years. Sixty-seven percent reported being in stable relationships.

The most accessed services were primary healthcare (PHC)(62%) followed by HIV (32%). Low proportions (≤6%) accessed STI,TB and malaria services. Most visits were characterised by only one service being accessed (93%, *n* = 15,523/16,688). Of the remaining 7% of visits, up to five services were accessed per visit and the combination of TB /HIV services in one visit remained extremely low (<1%, *n* = 14/16,688). Besides PHC services at the beginning of the reporting period, all service categories displayed similar seasonal utilisation trends(i.e. service utilisation peaked in the immediate few months post clinics opening and substantially decreased before holidays). Across all service categories, younger truck drivers, those with a stable partner currently, and those of South African origin were the main clinic attendees.

Older truck drivers (≥40 years) were more likely to access TB and PHC services, yet less likely to access HIV and STI services. Those with stable partners were less likely to access STI and TB services but more likely to access malaria and PHC services. South African attendees were more likely to access PHC, while attendees from other nationalities were more likely to access HIV and malaria services.

**Conclusions:**

This utilisation analysis shows that tailored services assist in alleviating healthcare access challenges faced by truck drivers, but it underscores the importance of ensuring that service packages and clinics speak to truck drivers’ needs in terms of services offered and clinic location.

**Electronic supplementary material:**

The online version of this article (10.1186/s12913-017-2595-3) contains supplementary material, which is available to authorized users.

## Background

Long distance truck drivers, due to their occupational circumstances, can be susceptible to worse health outcomes compared to the general population [1–6]. Research shows that truck drivers bear a disproportionate burden of both communicable (HIV, sexually transmitted infections (STI), tuberculosis (TB)) and non-communicable (chronic, mental, respiratory, cardiovascular, muscular) diseases [5, 7–13]. In particular, it is their potential contribution to the HIV epidemic [14–18] that has prompted countries and donors to support healthcare services specifically catering for this population [13]. As a result, existing healthcare programmes for truck drivers have introduced or re-oriented their services to HIV prevention and antiretroviral treatment, with a few programmes providing a broader variety of services. However, little is known about patterns of healthcare utilisation among truck drivers and whether this focus on HIV/AIDS is appropriate to client demand or whether other services should be provided, with these specialised programmes encouraged to expand their service provision.

An example of healthcare programmes for truck drivers is North Star Alliance, a non-governmental organisation providing healthcare to mobile populations and communities along transport corridors and at border crossings through a network of Roadside Wellness Centres (RWCs) across sub-Saharan Africa [19]. These RWCs are adapted shipping containers staffed with trained clinical and outreach teams. North Star Alliance provides a comprehensive menu of services, covering primary healthcare (PHC), HIV prevention and screening for a variety of infectious diseases (STI, TB, malaria…). With the exception of antiretroviral therapy (ART) services, the North Star Alliance RWCs in South Africa do not make use of paper-based client files; instead they are supported by a networked electronic data administration system called COMETS. This system was designed to facilitate the capture of clinical information, allowing clients to access their health records at every clinic within the network along transport corridors. In this study, we aim to describe healthcare utilisation among truck drivers across South Africa, using routinely collected data obtained by the COMETS system as well as to characterise clinic attendees to inform future service planning and development.

## Methods

### Setting

Nine RWCs across seven provinces in South Africa were included in this analysis. These clinics are located in Cato Ridge and Pongola (KwaZulu Natal), Bloemfontein (Free State), Bloemhof (North West), Ngodwana (Mpumalanga), Musina (Limpopo), Upington (Northern Cape), and Pomona and City Deep (Gauteng). Currently, eight of these clinics are operational. The City Deep site in the Gauteng province was closed due to challenges experienced by truck drivers in accessing the location and was relocated to another district. It now operates at the Pomona site (Gauteng province). North Star Alliance does not have RWCs in the remaining two provinces (Western Cape and Eastern Cape). In all RWCs, services are offered to truck drivers, sex workers and the immediate communities surrounding the RWCs.

### Data extraction

Anonymised data were last extracted on 11 April 2016 from North Star Alliance’s COMETS database for a two-year period from 1st October 2013 to 30th September 2015. Information extracted included site name, COMETS unique identification number, socio-demographic variables (such as age, sex, occupation, nationality and marital status) and visit information (including visit date and type of service accessed). Information such as educational level or income is not routinely collected in this system; therefore, this information could not be included in the analysis. After data extraction, we excluded duplicate and incomplete records. Records were then organised to provide both unique attendee and unique visit information.

### Analysis

We analysed all data using STATA 12. We restricted the analysis to a sub-set of records pertaining to truck drivers aged 18 to 65 years, as age could not be verified and there were some instances of unreliable information.

Continuous data (age) were described by mean and standard deviation (SD), then grouped into categories. Categorical data such as nationality and marital status were categorised in two groups each. For nationality, we defined South African and other nationalities. Other nationalities reported included a wide range of countries: Angola, Botswana, Congo and Democratic Republic of Congo, Ethiopia, Lesotho, Madagascar, Malawi, Mozambique, Namibia, Niger, Nigeria, Rwanda, Saint Helena, Senegal, Somalia, South Sudan, Sudan, Swaziland, Tanzania, Togo, Uganda, Zambia, and Zimbabwe. For marital status, we defined a group of truck drivers with no stable partner, including those who were currently divorced, single, or widowed; and a group with a stable partner, including those who reported being currently married or cohabiting.

We examined services accessed by type of service: HIV, STI, TB, malaria, and PHC. PHC comprises an array of services, including but not limited to respiratory, chronic, cardiovascular and muscular illnesses (more detail of services provided is given in Table S1 of the Additional file 1). We described the distribution by attendees and by visits across different socio-demographic characteristics and services accessed.

Finally, we assessed associations between services accessed and socio-demographic characteristics using univariable and multivariable logistic regression models. Robust standard errors were calculated for all models to allow for intragroup correlations and the sample structure, i.e. visits by individuals nested in sites. All variables were included in the multivariable models. We reported both unadjusted and adjusted odds ratios, 95% confidence intervals and *p* values. *P* values less than 0.05 were considered significant.

### Ethical considerations

Ethical clearance was provided by the University of the Witwatersrand Human Research Ethics Committee (M140506) on 30 May 2014. In addition, North Star Alliance and Wits Reproductive Health and HIV Institute signed a data user’s agreement on 5 May 2014 to ensure that data were appropriately handled throughout the data management cycle.

## Results

**Fig. 1:**
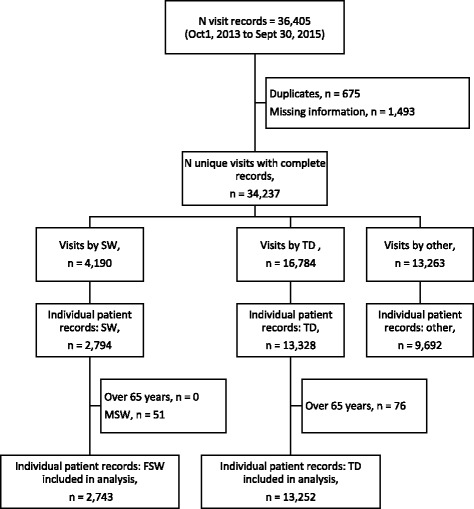
Flowchart of routinely collected data extraction and records selection. N, number; Oct, October; Sept, September; n, number; SW, sex worker; TD, truck driver; MSW, male sex worker; FSW, female sex worker

We extracted information for 36,405 records. After excluding duplicate records (*n* = 675) and incomplete records (*n* = 1493), we had 34,237 records. Of these, almost half (49%, 16,784/34,237) were records from truck drivers’ visits. After excluding truck drivers aged above 65 years, we observed 13,252 individual truck drivers who made 16,688 visits (an average of 1.26 visits/person) and accessed 17,885 services (an average of 1.07 services/visit and 1.35 services/person) across nine clinics during the 2 year reporting period (Fig. 1).

### Service utilisation

In Table 1, we present the distribution of individuals and services accessed by socio-demographic characteristics and type of services. The mean age of truck drivers accessing the sites was 39 years (SD 9,9). More than half (56%) of them were less than 40 years old (*n* = 7403/13,252), followed by 27% (*n* = 3551/13,252) in the 40–49 year age category, while less than 1% (*n* = 96/13,252) were younger than 20 years. Considering stability of partnerships, 67% (*n* = 8887/13,252) of truck drivers reported being either married (monogamy or polygamy) or cohabitating, with a large majority of these men being in a monogamous marriage (96%, *n* = 8575/8887). The majority of the truck drivers accessing services across the clinics was South African (82%, *n* = 10,825/13,252).

**Table 1:** Services accessed disaggregated by socio-demographic characteristics of truck drivers

		Services accessed (*n* = 17,885)				
	Clients	HIV, n (%)	STI, n (%)	TB, n (%)	Malaria, n (%)	PHC, n (%)
Total	13252	5660 (31.6)	1092 (6.1)	62 (0.3)	70 (0.4)	11001 (61.5)
Age, mean (SD)	39.2 (9.9)	37.2 (9.4)	36.2 (8.7)	40.4 (9.5)	39.2 (8.4)	41.1 (10.0)
Age						
< 20 years	96 (0.7)	59 (1.0)	11 (1.0)	0	0	45 (0.4)
20–29 years	2114 (16.0)	1131(20.1)	232 (21.2)	8 (12.9)	7 (10.0)	1330 (12.1)
30–39 years	5193 (39.2)	2470 (43.6)	511 (46.8)	22 (35.5)	36 (51.4)	3879 (35.3)
40–49 years	3551 (26.8)	1310 (23.1)	254 (23.3)	18 (29.0)	19 (27.1)	3273 (29.7)
50–65 years	2298 (17.3)	690 (12.2)	84 (7.7)	14 (22.6)	8 (11.4)	2474 (22.5)
Sex						
Female	182 (1.4)	68 (1.2)	14 (1.3)	1 (1.6)	0 (0.0)	119 (1.1)
Male	13070 (98.6)	5592 (98.8)	1078 (98.7)	61 (98.4)	70 (100.0)	10882 (98.9)
Marital status						
SP	8887 (67.1)	3807 (67.3)	639 (58.5)	39 (62.9)	61 (87.1)	7626 (69.3)
No SP	4365 (32.9)	1853 (32.7)	453 (41.5)	23 (37.1)	9 (12.9)	3375 (30.7)
Nationality						
South Africa	10825 (81.7)	4209 (74.4)	789 (72.3)	47 (75.8)	18 (25.7)	9373 (85.2)
Other	2427 (18.3)	1451 (25.6)	303 (27.7)	15 (24.2)	52 (74.3)	1628 (17.8)

*HIV* Human immunodeficiency virus, *STI* Sexually transmitted infection, *TB* Tuberculosis, *PHC* Primary healthcare, *n* Number, *No SP* No stable partner, *SP* With stable partner

The most accessed category of services provided by the clinics was PHC, accounting for 62% of the workload (*n* = 11,001/17,885). This was followed by HIV services at 32% (*n* = 5660/17,885). Lower proportions of services were accessed for STIs and less than 1% of services were accessed for TB and malaria, respectively. Across all service categories younger truck drivers, with a stable partner currently, and those of South African origin were the main clinic attendees.

**Fig. 2:**
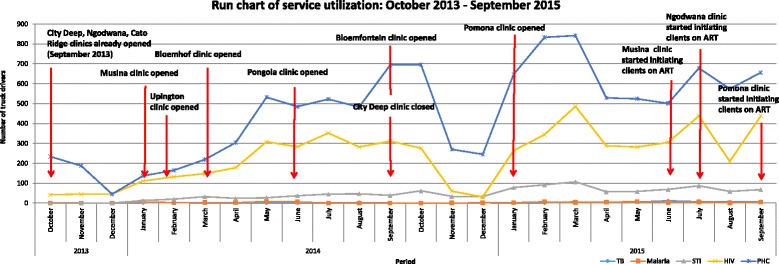
Service delivery and implementation for October 2013 to September 2015. ART, antiretroviral therapy

Figure 2 represents the variation across time of services accessed together with the opening of clinics. PHC services are consistently the type of service most accessed, with HIV services showing the same trends as PHC services over time. With the exception of PHC services at the beginning of the reporting period, all categories of services displayed the same trend of utilisation: service utilisation peaked in the immediate few months following the opening of new clinics, with a substantial decrease in demand seen in the period leading up to the end of year holidays. In the first fiscal year (from October 2013 to September 2014), 6569 services were accessed with an approximate 75% increase observed during the second fiscal year (from October 2014 to September 2015) in which 11,316 services were rendered to truck drivers.

**Fig. 3:**
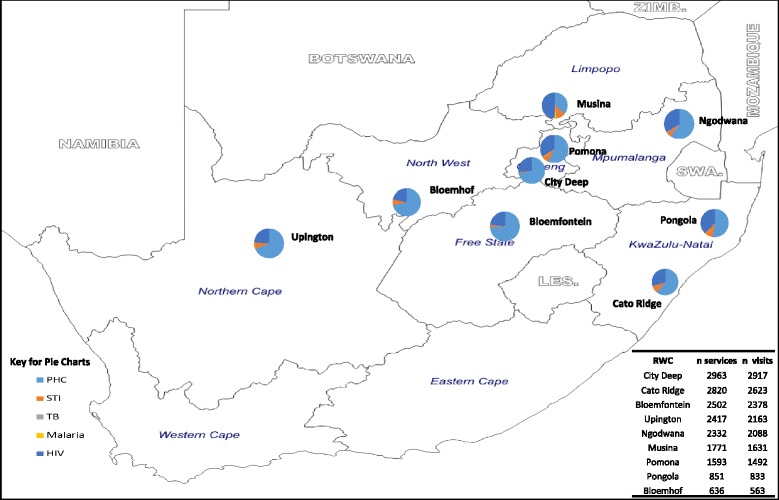
Map showing clinic location and service utilisation. PHC, primary healthcare; STI, sexually transmitted infection; TB, tuberculosis; HIV, human immunodeficiency virus; n, number

Figure 3 shows service utilisation for services by location. The majority of the sites served a similar volume of clients; of the operational facilities, the newest of the RWCs, Bloemfontein RWC, reported the highest headcount. Although there were challenges in providing services to truck drivers at the City Deep clinic, it reported the highest service volume (2963 services in 2917 visits). This was facilitated by healthcare workers providing mobile outreach services to truck drivers as opposed to truck drivers coming into the clinic. Clients of RWCs in high HIV prevalence border communities (e.g. Pongola; KwaZulu-Natal HIV prevalence = 28% [20]) or neighbouring high HIV prevalence areas (e.g. Musina (Limpopo) with a farm worker HIV prevalence = 28% [21]) accessed proportionally more HIV-related services, while other RWCs such as Bloemhof and Bloemfontein mainly dispensed PHC services.

The vast majority of visits saw only one service being accessed (93%, *n* = 15,523/16,688). Of the remaining 7% of visits, up to five services were accessed per visit. The most sought after multiple service combination was PHC and HIV (5%, *n* = 835/16,688) followed by PHC and STI (less than 1%, *n* = 145/16,688). The combination of TB and HIV services in one visit remained extremely low at less than 1% (*n* = 14/16,688).

### Factors associated with service utilisation

In Table 2, we present the results of both univariable and multivariable analyses. Older truck drivers were more likely to access PHC services and less likely to access TB, HIV or STI services. Those with stable partners currently were less likely to access STI and TB services than those without a stable partner currently. South African attendees were more likely than non-South Africans to access PHC, while attendees from other nationalities were more likely to access HIV and malaria services.

**Table 2:** Determinants of service uptake across all sites

	HIV (*n* = 16,688 records; *n* = 5660 services)		STI (*n* = 16,688 records; *n* = 1092 services)		TB (*n* = 16,580 records^a^; *n* = 62 services)		MALARIA (*n* = 16,580 records^a^; *n* = 70 services)		PHC (*n* = 16,688 records; *n* = 11,001 services)	
Age	UOR [95% CI]	AOR [95% CI]	UOR [95% CI]	AOR [95% CI]	UOR [95% CI]	AOR [95% CI]	UOR [95% CI]	AOR [95% CI]	UOR [95% CI]	AOR [95% CI]
<20 years	reference	reference	reference	reference	–	–	–	–	reference	reference
20–29 years	0.67 [0.25–1.84]	0.67 [0.23–1.92]	0.89 [0.50–1.58]	0.82 [0.45–1.48]	reference	reference	reference	reference	1.56 [0.55–4.44]	1.62 [0.53–4.92]
30–39 years	0.52 [0.18–0.48]	0.49 [0.17–1.38]	0.76 [0.43–1.36]	0.75 [0.42–1.34]	1.08 [0.41–2.82]	0.91 [0.72–1.16]	2.03 [0.66–6.24]	1.59 [0.46–5.46]	2.14 [0.78–5.86]	2.30 [0.83–6.36]
40–49 years	0.34 [0.13–0.84]*	0.31 [0.13–0.77]*	0.52 [0.28–0.98]*	0.56 [0.30–1.03]	1.25 [0.54–2.88]	0.68 [0.52–0.89]**	1.50 [0.61–3.71]	1.30 [0.46–3.62]	3.56 [1.46–8.70]**	3.75 [1.54–9.12]**
50–65 years	0.24 [0.09–0.65]**	0.24 [0.09–0.63]***	0.25 [0.13–0.49]***	0.29 [0.15–0.57]***	1.44 [0.63–3.28]	0.35 [0.22–0.58]***	0.94 [0.29–3.02]	1.24 [0.40–3.89]	5.76 [2.32–14.30]***	5.62 [2.30–13.72]***
Marital status										
No SP	reference	reference	reference	reference	reference	reference	reference	reference	reference	reference
SP	0.95 [0.65–1.37]	1.10 [0.76–1.60]	0.64 [0.42–0.98]*	0.68 [0.57–0.81]***	0.80 [0.55–1.15]	0.68 [0.57–0.82]***	3.19 [1.89–5.40]***	1.54 [0.84–2.81]	1.18 [0.78–1.81]	1.01 [0.73–1.41]
Nationality										
South African	reference	reference	reference	reference	reference	reference	reference	reference	reference	reference
Other	1.87 [1.18–2.96]**	1.72 [1.13–2.60]*	1.70 [0.92–3.14]	1.77 [0.95–3.28]	1.36 [0.70–2.65]	1.72 [0.92–3.23]	12.50 [4.44–35.19]***	11.04 [3.89–31.29]***	0.47 [0.25–0.88]*	0.50 [0.27–0.90]*

*HIV* Human immunodeficiency virus, *STI* Sexually transmitted infection, *TB* Tuberculosis, *PHC* Primary healthcare, *n* Number, *UOR* Unadjusted odds ratio, *AOR* Adjusted odds ratio (refers to adjusted for all variables in table); 95% CI, 95% confidence interval; No SP: no stable partner includes the following categories: divorced, single or widowed. SP: with stable partner includes married and cohabiting. *indicates a *p* value < 0.05; **p value < 0.01; ***p value < 0.001

^a^records with <20y (*n* = 108) were dropped in this analysis, because there were no TB/malaria visits recorded

## Discussion

To our knowledge, this is the first report of comprehensive health service utilisation among truck drivers in South Africa. Our main results show that truck drivers attending RWCs are mainly middle-aged and in stable relationships. While HIV services are important to truck drivers from other countries, PHC services are most in demand among South African truck drivers. It could be argued that North Star Alliance’s comprehensive service package suits the majority of South African clinic attendees and that further development of PHC services might be an important consideration for future healthcare planning in South Africa.

As anticipated, as the number of RWCs increased over time, the absolute number of clients accessing services increased. However, changes in service delivery such as increased efforts to promote the clinics through outreach, the introduction of ART services for HIV-positive clients, and the increase in scope of the PHC services offered are likely to have contributed to increasing service uptake. The same yearly patterns of utilisation overall were mirrored across service type, i.e. peaks during the second quarter of the year and troughs in the last quarter. This pattern of utilisation is in keeping with trends seen among other service providers in the South African public healthcare system. The last quarter decreases are usually attributed to companies slowing down work as the year end approaches and closing for holidays.

STI prevalence in the truck driver population has been estimated to be higher than in the general population [22]. Our results showed that STI service utilisation in this mobile population was low. However, this might be related to the profile of clinic attendees (i.e. older and in stable partnerships) who might be at lower risk.

The low malaria service utilisation could be explained by South Africa’s low malaria prevalence [23] and resulting low risk perception. While HIV services were second highest in demand, the TB and HIV combination remained extremely low (less than 1%). Although the entry point for services cannot be determined from the database, it is apparent that there is an opportunity to improve provider-initiated testing and counselling [24] services and to promote screening for multiple diseases in a single visit, such as TB screening for HIV clients and vice versa but also in combination with non-communicable disease screening.

Lastly, we observe different patterns of utilisation, both in volume and type of services, depending on location. Although the Bloemfontein clinic was one of the last clinics to become operational, it had the highest number of people accessing the clinic because it is located on a busy national route and offers the most potential for service utilisation and uptake. The absence of RWCs in Western and Eastern Cape does not allow us to comment on truck driver healthcare utilisation in those provinces. However, based on the arrangement of the South African trucking corridor, the Bloemfontein RWC could be used as a proxy for utilisation trends. From the geographical size and infrastructure perspectives, we expect that truck driver utilisation would be much higher in the Western Cape as compared to the Eastern Cape.

Our characterisation of clinic attendees indicates that the cross-border mobile population was mostly likely to access services for communicable diseases, such as malaria and HIV services, whereas South African drivers were most likely to access PHC services. This could imply that transiting truck drivers may feel more comfortable accessing communicable disease services outside their home country while on the road, with North Star Alliance possibly filling a gap in service provision for this population. Programmatically this begs the question of whether non-South Africans access services in this way to avoid stigma, discrimination, and unnecessary disclosure in their home countries – especially in the case of positive results for HIV and STIs. However, linkage to care and treatment for them remains a challenge.

North Star Alliance also provides services to local communities both in Zambia and Zimbabwe. However, routinely collected data from other countries show that patients in South Africa tend to come to North Star Alliance services more regularly than in the other countries. There is a similar pattern of utilization by truck drivers both by country and over time. In South Africa, truck drivers tend to attend PHC services and these visits have increased over the two-year period. In Zambia, truck drivers mostly access HIV-related services while in Zimbabwe, they access both PHC and HIV-related services. The numbers of services accessed have remained stable in Zambia and Zimbabwe. Albeit the variable data quality across countries utilisation is more likely to be attributed to the overall healthcare services, facilities and general infrastructure available to the general populations in Zambia and Zimbabwe.

Formally contextualising and generalising findings from this study to the African trucker population is limited by the dearth of available comprehensive truck driver healthcare utilisation data; possibly linked to services offered to them (the emphasis on sexually transmitted diseases in truck drivers has influenced health programming for truck drivers to focus on HIV (behaviour change communication, outreach, condom distribution and testing) and STIs) and programmes’ general lack of recording and reporting [13]. However a Nigerian survey reported that commercial truck drivers in the age range 30–39 years were most likely to use HIV services [25], where we found no significance in our study. Moreover, a survey conducted in the US among long distance truck drivers similarly found that the middle aged, married truck drivers had the most interest in PHC related services and less interest in STIs; dissimilarly finding that HIV was the second least of interest [5]. Accessing healthcare is in part related to availability and this availability is related to a country’s evidenced or perceived burden of diseases. From the observations mentioned above it stands to reason that high burdened HIV countries will have a strong HIV response and healthcare utilisation will be favoured towards HIV services. However, it is crucial to note that lack of reporting hinders the development of appropriate country responses which ultimately influences patterns of utilisation, uptake and health outcomes not only in truck drivers but in all populations (priority and general).

This study presents some limitations. Firstly, with routinely collected information only, there is the possibility that utilisation is actually higher than that reported and misreporting is possible. However, when paper records were also available, data quality checks were performed periodically as part of quality assurance programmes for external funders. Secondly, the data describe healthcare utilisation among attendees of North Star Alliance’s RWCs and thus exclude both truck drivers who require health services but have not been accessing them at all and those accessing services from providers other than RWCs. Nevertheless, our results highlight the need for programme planners to consider the following vital aspects when designing healthcare packages for this priority population. Firstly, given that truck drivers mostly access services for non-communicable diseases, the lack of provider-initiated testing and counselling illustrates an important missed opportunity. Comprehensive care service delivery packages and training for staff to deliver them in these settings will increase the quality and impact of services available to truck drivers, which in turn might increase service demand. Attracting more truck drivers into the clinics provides further opportunities for healthcare providers to offer screening, testing, and/or management of other communicable diseases. This comprehensive care and its increased use will simultaneously generate a healthier truck driver population, with subsequent economic benefits, and will address diseases that are public health priorities.

## Conclusion

This utilisation analysis shows that tailored services do assist in alleviating healthcare access challenges faced by truck drivers. It is however important to ensure that service packages and clinics speak to the needs of truck drivers in terms of services offered and clinic location. A comprehensive package of care will tackle both communicable and non-communicable disease burdens and provide South Africa with a healthier truck driver workforce.

## Additional file

Additional file 1: Lalla-Edward_ Sup mat S1_Uptake of health services. **Table S1.** North Star Alliance Tier I core service package implementation guidelines. Narrative description of core services/service categories offered by North Star Alliance Roadside Wellness Centres. (DOCX 38 kb)

## Abbreviations

AIDS: Acquired Immune Deficiency Syndrome
AOR: Adjusted Odds Ratio
ART: Antiretroviral Therapy
CI: Confidence Interval
COMETS: Corridor Medical Transfer System
HIV: Human Immunodeficiency Virus
n: Number
PHC: Primary Healthcare
RWC: Roadside Wellness Centre
SD: Standard Deviation
SP: Stable Partner
STI: Sexually Transmitted Infection
TB: Tuberculosis
UOR: Unadjusted Odds Ratio
