# Supplementary material for: Uptake of health services among truck drivers in South Africa: analysis of routine data from nine roadside wellness centres
Source: BMC Health Serv Res. 2017 Sep 13;17:649. doi: 10.1186/s12913-017-2595-3 (PMC5598062; doi:10.1186/s12913-017-2595-3)
Supplement: Additional file 1: — Lalla-Edward_ Sup mat S1_Uptake of health services. Table S1. North Star Alliance Tier I core service package implementation guidelines. Narrative description of core services/service categories offered by North Star Alliance Roadside Wellness Centres. (DOCX 38 kb) [file 12913_2017_2595_MOESM1_ESM.docx]

Additional file 1

Table S1: North Star Alliance Tier I core service package implementation guidelines.

|  | **PREVENTION** | **SCREENING** | **DIAGNOSIS AND TESTS** | **CARE AND TREATMENT** | **REFERRAL** |
| --- | --- | --- | --- | --- | --- |
| **HIV/AIDS** | Health promotion and education; Condom demonstration and distribution; Male circumcision; Outreach/moonlight; PREP; PEP | Medical history; Sexual behaviour; STIs; Blood transfusion; Previous HCT; and any other risk factors;  Physical examination (Symptoms of OIs, STIs, TB ) | HIV rapid Testing (*follow user instruction)*; advise on return visit/ window period, on partner’s testing;  CD4 (rapid lab test – Pima machine);  VL | Nutritional supplements; Basic care of OI; Side effects of ART ; Emergency provision of ART; Cotrimoxazole; Counselling: adherence and disclosure to support person | **To referral hospital /team** for complementary and periodic investigations; Nutrition and psychological support; Second line treatment; Management of severe side effects |
| **STIs** | Health promotion and education; Condom demonstration and distribution; Male circumcision; Outreach/moonlight; Partner contact | Personal medical history; Behavioural risk factors; Physical examination | Syphilis rapid test(*follow user instruction)*; Provider initiated HCT | **Syndromic management**  Counselling for partner treatment; Advise on follow up and return date | **To referral hospital** for recurrence, non-responsive and complicated cases |
| **TB** | Health promotion and education; Infection control measures in facility (ventilation / N95 masks); INH prophylaxis | Personal medical history; Socio and professional background; Physical examination; Screening questionnaire | Weight/BMI; Provider initiated HCT; Sputum collection for symptomatic clients | Counselling on adherence to treatment and periodic follow-up for treated cases | **To referral hospital** for further investigations (Chest X-ray) |
| **MALARIA** | Health promotion and education (excl. vector control at domestic level); Provision of preventive/ prophylactic treatment to traveller | Medical history; Personal social and professional background; Current treatment (over-the-counter, traditional etc.); Travel history | Physical examination; Malaria rapid testing | Provide malaria and symptomatic treatments; Advise on second course of treatment one week later in high incidence countries and on preventive actions | **To nearest hospital** for complicated cases ( neurological, vomiting, uncontrolled temperature >40°C) |
| **GASTRO -INTESTINAL INFECTIONS / DISORDERS** | Health promotion and education (personal and food hygiene ); Public health education and sensitization  Dietary advice | Medical history (recurrence, food and drink hygiene, habits, HIV status); Current treatment (over-the-counter, traditional etc.); Physical examination ( dehydration ) | If recurrence, propose rapid HIV testing | Antibiotics; Probiotics or anti-fungals where indicated; ORS provided in mild dehydration; Anti-spasmodics; Antacids for symptomatic relief | **To nearest hospital** for continuous vomiting, severe dehydration; suspected notifiable diseases |
| **RESPIRATORY TRACT INFECTIONS** | Health promotion and education**;** Public health education and sensitization | Medical history (HIV status); Personal social background (close contact with TB cases); Current treatment (over-the-counter, traditional etc.); Physical examination; TB screening questionnaire | If suspect of TB: Sputum collection**,** Propose HIV test | Antibiotics when evidence of microbial infection or community acquired pneumonia; Mucolytics and bronchodilators where indicated | **To referral hospital** **reachable in next 48 hours**  for complementary investigations (chest X-ray); **To nearest hospital** for severe or life threatening cases |
| **EAR, NOSE AND THROAT AND EYE INFECTIONS** | Health promotion and education**;** Public health education and sensitization | Medical history ( recurrence );  Physical examination | Snellen test for visual accuracy; ENT exam; Ortoscope for ears; Visual assessment of larynx and under light | Antibiotics, anti-inflammations, antihistamines | **To specialist** if recurrence, chronic, severe |
| **SKIN INFECTIONS & DISORDERS** | Health promotion and education (allergens, personal hygiene); Public health education and sensitization | Medical history; Socio-economic; Professional background; Physical examination | Aspect of lesions (diagnosis supported by visual documentation); Physical exam | **Symptomatic and etiologic management**  Topical antifungals / antibiotics / steroids where indicated | **To specialist** for recurrence, chronic and severe cases |
| **HYPERTENSION** | Health promotion and education (lifestyle and nutrition) | Medical history - personal, familial;  Professional background; Current treatment (over-the-counter, traditional etc.); Physical exam (cardiac) | BP, Weight, BMI, urine test *(follow user instruction)*; Repeat BP measure after rest; Classify / stage for treatment | Life style modification counselling; Anti-hypertensives according to stage; Fast acting anti-HPT for acute/ severe cases | **To nearest hospital** if not controllable at RWC; for further investigation and in case HPT urgency and emergency |
| **DIABETES** | Health promotion and education (lifestyle and nutrition) | Medical history - personal, familial;  Professional background (over-the-counter, traditional etc.) ;  Physical examination (signs of associated illnesses ) | Urine test; Blood test; Weight , BMI;  *Refer to related SOPs*  *(Physical exam – exclude complications / target organ damage)* | Nutrition counselling if mild; Treatment initiation for complications / mild cases; Maintaining and follow-up compulsory for treatment modification | **To nearest hospital** if secondary complications target organ damage |
| **CERVICAL CANCER** | Health promotion and education**;** Public health education and sensitization; Promote health seeking behaviour - immunization or periodic medical screening according to age group | Medical history (STIs, pain, bleeding); Physical examination  **Preventive screening**:  All female STI cases; All women from age 25 to 50 | N/A | N/A | **To referral hospital** for pap smear and other investigations |
| **BONE AND JOINT DISORDERS** | Health promotion  and education **(**ergonomic, workplace station organization, manual handling ) | Personal medical history; Professional background and tasks; Former and current treatment | Physical examination | Analysis / anti-inflammatory (calcium supplements) (where indicated) | **To specialist** for investigations and care; Blood tests (autoimmune diagnosis; rheumatoid diagnosis);  Orthopedic consultation and radiology |
| **NEURO -PSYCHOLOGICAL DISORDERS** | Health education  (Stress factors – stress mitigation) | Personal medical history (meningitis, depression, epilepsy); Professional background & tasks; Physical examination; Former and current treatment; Substance abuse; Check for stress-related condition | Vital signs; CNS physical exam; Mini mental state exam | **Symptomatic management:**  Analgesics / anti-inflammatories | **To nearest hospital** if severe or uncontrollable; **To specialist** for further investigations and/or care  If relevant, advise for psychological support |
| **SUBSTANCE USE DISORDER** | Health promotion  and education; Public health education and sensitization; Lifestyle modification | Identification of substance **and** of substance use disorder: complaints, observation, questions, relationship with other organic and systemic health conditions; (Consider illegal drugs, medicines, traditional “drugs”, alcohol); Physical examination | HIV rapid test in case of PWID | Education and counselling; Symptomatic care; Forced rest in case where behaviour is incompatible with driving, risky behaviour for self and others | **To nearest referral** **hospital** for severe intoxication; **To specialist** for psychological and medical support and for detoxification |
| **EMERGENCY / FIRST AID** | Health education and sensitization (domestic and “on the road” safety);  Emergency contact list, local and along corridor | Physical examination: Rapid primary and secondary assessment of case severity Rapid screening for referral | First aid / basic life support techniques / vitals monitoring / fully equipped emergency trolley | First aid measures for full recovery or stabilization prior to referral, in a timely manner | **To nearest referral hospital** for further care, investigations and/or follow up (EMRS services used) |
| **FAMILY PLANNING** | Health promotion and education**;** | Pregnancy test prior  HIV test offered | N/A | Injectable contraceptive (2 and 3 month doses); Oral contraceptives ;  Male and female condoms | **To** **local family planning services for other forms of contraception** |

HIV, human immunodeficiency virus; AIDS, acquired immune deficiency syndrome; PREP, pre-exposure prophylaxis; PEP, post exposure prophylaxis; STI, sexually transmitted infection; HCT, HIV Counselling and Testing; OI, opportunistic infection; TB, tuberculosis; VL, viral load; ART, antiretroviral therapy; INH, isoniazid; BMI, body mass index; etc., etcetera; ENT, ear, nose and throat; BP, blood pressure; RWC, roadside wellness centre; HPT, hypertension; SOP, standard operating procedure; NA, not applicable; CNS, central nervous system; PWID, person who injects drugs; EMS, emergency medical services
